# Evaluating mFARS in pediatric Friedreich's ataxia: Insights from the FACHILD study

**DOI:** 10.1002/acn3.52057

**Published:** 2024-03-31

**Authors:** Christian Rummey, Susan Perlman, Sub. H. Subramony, Jennifer Farmer, David R. Lynch

**Affiliations:** ^1^ Clinical Data Science GmbH Basel Switzerland; ^2^ Department of Neurology University of California Los Angeles California USA; ^3^ Department of Neurology Fixel Center for Neurological Disorders 3009, SW Williston Road Gainesville Florida 32608 USA; ^4^ Friedreich's Ataxia Research Alliance Downingtown Pennsylvania USA; ^5^ Departments of Pediatrics and Neurology Children's Hospital of Philadelphia Philadelphia Pennsylvania USA

## Abstract

**Objectives:**

Friedreich ataxia (FRDA) is a rare genetic disorder caused by mutations in the *FXN* gene, leading to progressive coordination loss and other symptoms. The recently approved omaveloxolone targets this condition but is limited to patients over 16 years of age, highlighting the need for pediatric treatments due to the disorder's early onset and more rapid progression in children. This population also experiences increased non‐neurological complications; the FACHILD study aimed to augment and expand the knowledge about the natural history of the disease and clinical outcome assessments for trials in children in FRDA.

**Methods:**

The study enrolled 108 individuals aged 7–18 years with a confirmed FRDA diagnosis, with visits occurring from October 2017 to November 2022 across three institutions. Several measures were introduced to minimize the impact of the COVID‐19 pandemic, including virtual visits. Outcome measures centered on the mFARS score and its subscores, and data were analyzed using mixed models for repeated measures. For context and to avoid misinterpretation, the analysis was augmented with data from patients enrolled in the Friedreich's Ataxia Clinical Outcome Measures Study.

**Results:**

Results confirmed the general usefulness of the mFARS score in children, but also highlighted issues, particularly with the upper limb subscore (FARS B). Increased variability, limited homogeneity across study subgroups, and potential training effects might limit mFARS application in clinical trials in pediatric populations.

**Interpretation:**

The FARS E (Upright Stability) score might be a preferred outcome measure in this patient population.

## Introduction

Friedreich ataxia (FRDA) is a rare hereditary condition marked by a continuous decline in coordination and stability, accompanied by multiple additional manifestations. It arises due to alterations in the *FXN* gene responsible for generating frataxin, an essential protein for mitochondrial activity. Most commonly diagnosed in children or teenagers, FRDA progressively intensifies, leading to considerable impairment and a shortened lifespan. Until very recently, treatment options for FRDA were limited to addressing its symptoms.

The recent approval of omaveloxolone^1^, ^2^ offers a path forward for clinical development while also raising the need for trials in children with FRDA. The pivotal study used the modified Friedreich Ataxia Rating Scale (mFARS) as the primary outcome measure, establishing it as a clinician reported outcome measures for assessing disease status and progression in ataxia. Based on the population studied, omaveloxolone has received approval solely for patients aged 16 years and above. This emphasizes the need for pediatric trials, especially considering the early manifestation and profound progression in children with FRDA. Ideally, therapeutic interventions should be initiated at the earliest opportunity.

Children, defined here as individuals aged less than 18 years, with FRDA are the most severely affected patient group, with specific differences compared to adult or later onset populations, despite sharing an essentially identical neurological phenotype. They experience faster disease progression but also the prevalence of non‐neurological features, including cardiac complications, diabetes, and scoliosis increases dramatically with younger age of onset. Consequently, it remains unclear if all specific functions captured by the mFARS can be assessed reliably in children, raising the possibility that mFARS might not have the same sensitivity in children compared to adults.

The Friedreich's Ataxia Clinical Outcome Measures Study (FACOMS)^3^ is to date the largest natural history study in FRDA and has enrolled patients of all ages continuously since 2003. Due to this study, neurological progression in FRDA, as assessed by the mFARS, is today relatively well understood. On the other hand, the relative absence of particularly the youngest children is one of FACOMS' weaknesses and clinical outcome measures were not adapted specifically to children. In addition, FACOMS assessments are relatively infrequent (once per year) and therefore produce limited amounts data compared to typical clinical trials.

Few studies have been conducted in children, and no specific (e.g., subgroup) results exist to describe in detail this population. The present FACHILD study is an effort to address these gaps, by focusing on pediatric subjects, including biannual assessments, and evaluation of new measures more relevant to children (including patient reported outcome measures). This manuscript focuses on the mFARS examination and contextualizes the results with a comparable subgroup from FACOMS.

## Materials and Methods

### Participants and populations

The present study enrolled 108 males or females aged 7–18 years with a genetically confirmed diagnosis of FRDA (by protocol, ages 2 years and higher were allowed). Baseline visits took place between October 2017 and October 2019, and the last patient visit in November 2022. Seventy‐seven subjects (71%) were evaluated at the Children's Hospital of Philadelphia, 18 (17%) at the University of California, Los Angeles, and 13 (12%) at the University of Florida, Gainesville. All individuals registered for FACHILD also participated in FACOMS. FACHILD was registered at clinicaltrials.gov as NCT03418740.

FACHILD was designed to capture a representative sample of the complete pediatric FRDA population and included outcome measures for later stage individuals as well as nonfunctional biomarkers. Ataxia rating scales, however, are only sensitive to change in ambulatory populations^4^, ^5^, and some individuals therefore would not pass typical inclusion criteria for concurrent clinical trials (mFARS baseline threshold and/or retainment of a certain capacity to walk, such as walking 10 meters without assistance). Accordingly, nonambulatory individuals were excluded from this analysis (assessed by functional disease stage, FDS ≥5, that is, full‐time wheelchair bound).

The intention of using a FACOMS comparison group was not to match a most similar cohort (as in a propensity matching or natural history control study), but to be as inclusive as possible based on the same selection criteria. FACOMS participants were selected for this work if they (1) were within the same age and mFARS range at baseline, (2) had at least a baseline and one follow‐up FACOMS visit within 3 years, and (3) had not participated in FACHILD.

### Outcome measures

The total mFARS score and its subscores were evaluated as outcome measures. The mFARS is composed of FARS A (bulbar subscore), FARS B (upper extremity), FARS C (lower limbs), and FARS E (balance and gait; FARS E is also called the upright stability score, USS). The contribution of FARS A (bulbar) to total change over time is minimal (yearly rate of change <0.1 in all published populations)^4^ and is not reported separately. In subsequent sections, all subscales are referred by this letter designation (FARS B, C, and E).

### Statistical analyses

Typical demographic parameters, as well as virtual visits and missing data were summarized descriptively. Serial observations were analyzed using mixed models with repeated measures (MMRM) including all data as available without data imputation. We used change from baseline as the dependent variable, and baseline value (continuous) and visit (fixed factor) as covariates. In separate analyses, the impact of potential covariate candidates (age of onset, the shorter GAA repeat length, baseline age) on change from baseline was investigated. We have recently discussed the relevance of population ages and proper stratification in clinical trials in FRDA^4^, with population age and patients' functional status being the most important predictors of progression. To assess these potential heterogeneities, all outcomes were analyzed within respective subgroups (median age, median baseline mFARS, and median baseline FARS E); FACHILD median values were used as thresholds in both cohorts. There were no prespecified hypotheses; statistical tests were performed two‐sided and interpreted in a descriptive, exploratory way with *p*‐values <0.05 considered statistically significant. Statistical calculations were performed in R (R Core Team, 2023) using lme4^6^ for mixed‐effect models.

### Standard protocol approvals, registrations, and patient consent

Written informed consent was obtained from all patients or their authorized surrogates at enrollment and renewed at every yearly visit. The study was approved by the local ethics committee of each participating center and is registered as NCT03418740 with clinicaltrials.gov.

## Results

### Visit structure and impact of the COVID‐19 pandemic

**Figure 1:**
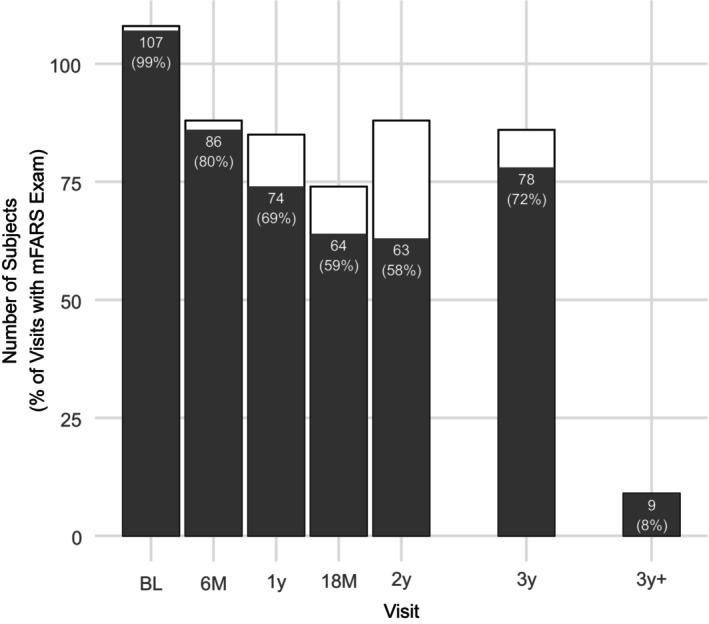
Overview of visits performed and available mFARS examinations. mFARS examinations can only be performed at in‐person visits. At the 3 years timepoint, 78 FARS examinations (72%) were available.

The study was designed with repeated visits every 6 months for 2 years, with one additional visit at 3 years. The onset of the COVID pandemic affected our ability to collect follow‐up data as planned. As a countermeasure, virtual visits were introduced as quickly as March 2020, which unfortunately did not allow the administration of the mFARS score (see white bars in Fig. 1). Nevertheless, 481 (89%) of 538 total visits had complete mFARS data available, including 78% of the final 3 years visits. This was facilitated due to restrictions being lifted during 2021 and 2022, when all patients were re‐summoned for a final closure visit. Nine participants (8%) returned for the final visit but missed the 6‐month visit window for year 3 (Fig. 1). Twenty‐three individuals (21%) withdrew from the study before the final visit; 13 lost to follow‐up, 9 unwilling/unable/related to schedule and pandemic/traveling issues. One was unable to continue consent. A chronological visit overview is presented in Figure S1.

### Demographic and baseline results

The population enrolled in this study was a young, severely affected cohort, indicated by high GAA1 repeat lengths and early ages of onset (Table 1). In the context of a previously suggested categorization,^4^ it was a mixed early/typical FRDA population, suggesting a certain level of diversity despite tight pediatric enrollment criteria. FACHILD did have a high number of participants who are compound heterozygous for the GAA repeat (8, 9.0%).

**Table 1:** Demographic characteristics of the analysis populations in FACHILD and the comparative FACOMS cohort. Numbers after the scales indicate maximum possible score.

	FACHILD	FACOMS
*N*	89	298
Sex (male, %)	41 (46.1%)	163 (54.7%)
Age	13.3 (2.8)	13.2 (3.0)
Age of onset	7.0 [5.0, 10.0]	8.0 [5.0, 11.0]
GAA1	766.0 [699.0, 893.8]	750.0 [650.0, 856.0]
GAA2	1000.0 [927.0, 1129.8]	950 [833.0, 1057.0]
Compound Heterozygotes	8 (9.0%)	20 (6.7%)
mFARS total (93)	37.8 (11.7)	40.6 (11.0)
Upright stability (FARS E, 36)	22.3 (5.3)	21.7 (5.6)
Upper limbs (FARS B, 36)	9.2 (5.3)	12.0 (5.0)
Lower limbs (FARS C, 16)	5.9 (2.4)	6.4 (2.8)
Bulbar function (FARS A, 5)	0.3 (0.5)	0.6 (0.6)

Data are *n* (%), median [IQR] or mean (SD).

As discussed above, individuals had to be ambulatory (FDS <5) and have at least one follow‐up visit. The typical mFARS threshold (20) for inclusion was relaxed to allow individuals with baseline scores between 17 and 19 to be included. Overall, among 108 individuals enrolled, 19 were excluded from the analysis population for one or more reasons (Table S1): Missing mFARS data (baseline or follow‐up, *n* = 6), early disease stage (*n* = 3, mFARS baseline values 1, 7, 12.3), and inability to ambulate at baseline (*n* = 13).

In the analysis population, mean baseline age was 13.3 years (SD 2.8), and mean mFARS score was 37.8 points (SD 11.7). The FACOMS comparison cohort was larger (*n* = 298) with enrollment spanning the complete FACOMS range (starting in 2003). It had similar features with slightly higher mFARS Total scores (Table 1), resulting from higher FARS B scores (9.2 in FACHILD vs 12.0 in FACOMS). Baseline FARS A was 0.3 points (SD 0.5) and 0.6 points (SD 0.6) at FACHILD and FACOMS respectively, indicating minimally impaired bulbar function and contributing less than 1% to total scores.

**Figure 2:**
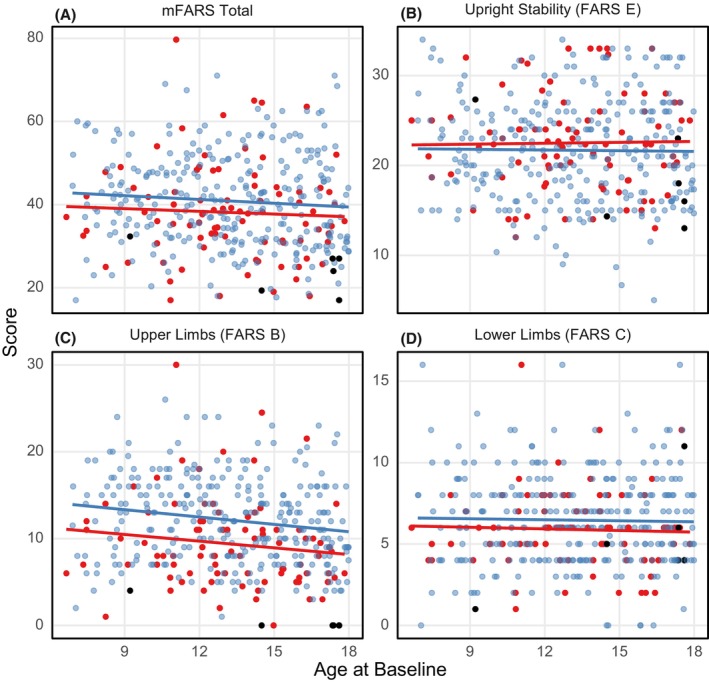
mFARS scores at baseline. FACHILD/FACOMS scores are shown in red/blue, respectively (analysis population). Black dots indicate individuals carrying G130V/154F mutations (see text for specifics).

Baseline mFARS scores in both FACHILD and FACOMS correlated only minimally with age (Fig. 2). Upper limb function, as previously suggested,^4^ was more affected in younger children, resulting in a negative age/baseline FARS B correlation (Fig. 2C). These results demonstrate the wide variation of both clinical status and genetic severity within children with FRDA. Patients compound heterozygous for the *FXN* mutations were included in both FACHILD and FACOMS (*n* = 8, 9.0% and *n* = 19, 6.5%, respectively).

The most frequently found pathogenic mutation in FRDA patients who are compound heterozygous is G130V.^7^ Together with the I154F mutation, it usually causes a specific phenotype with little to no upper limb involvement, while balance and gait issues are consistent with the common FRDA progression.^7^ Both cohorts included a total of five patients with G130V and one with I154F (FACOMS). Of those, one patient (aged 9) had a baseline FARS B score of 4, and all others (ages 14, 17, 17, 18, 18) scored 0 on FARS B. These observations were excluded from the FARS B/Age correlation in Figure 2.

### Analyses of yearly changes

**Figure 3:**
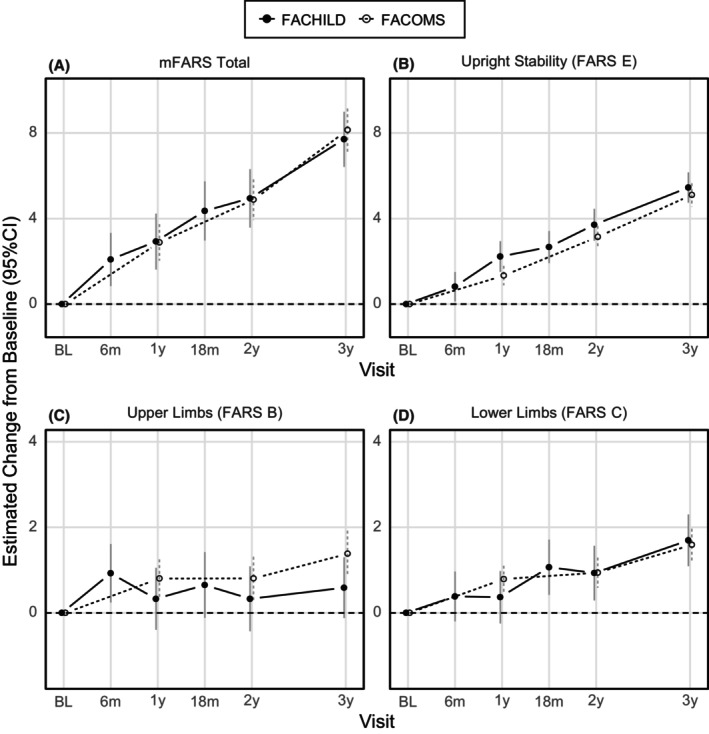
Changes from baseline in mFARS and its subscores over 3 years, for FACHILD (solid dots and lines) and FACOMS (circles, dotted lines).

The mFARS score captured well the rapid disease progression in children with FRDA. The overall change after 3 years was 7.7 points (95% CI 6.4 to 9.0), corresponding to a yearly rate of decline of 2.6 points. This rate was remarkably similar throughout the study period (Fig. 3A, numerical results are summarized in Table S2). The FACOMS cohort declined slightly faster (8.1 points at 3 years, 95% CI 7.1 to 9.1). The increase in mFARS over time was driven by FARS E (balance and gait), and to a smaller extent, FARS C (lower limbs). Over the 3‐year study period, changes in FARS E were similar (Fig. 3B) but not identical between the cohorts (FACHILD 5.5, 95% CI 4.7 to 6.2 vs. FACOMS 5.1, 95% CI 4.5 to 5.6). Differences between both studies were more apparent in FARS B: FACHILD participants progressed faster during the first 6 months and while fluctuating, stayed relatively stable throughout the rest of the study (up to 3 years, Fig. 3C). On the other hand, although FARS B decline in FACOMS seemed initially slower, it was later consistently higher at all three time points.

These differences impacted the sensitivity to detect change of mFARS, as assessed by standard response means (SRM, that is, descriptive mean change from baseline divided by the standard deviation of change). For the most relevant 1‐year timepoint and in FACHILD, FARS E alone was more sensitive than mFARS (SRM 0.77 vs 0.57, respectively). On the other hand, in FACOMS, total mFARS was the more sensitive outcome over FARS E (SRM 0.43 vs 0.33 respectively). Yearly SRMs for these outcomes are summarized in Table S2.

**Figure 4:**
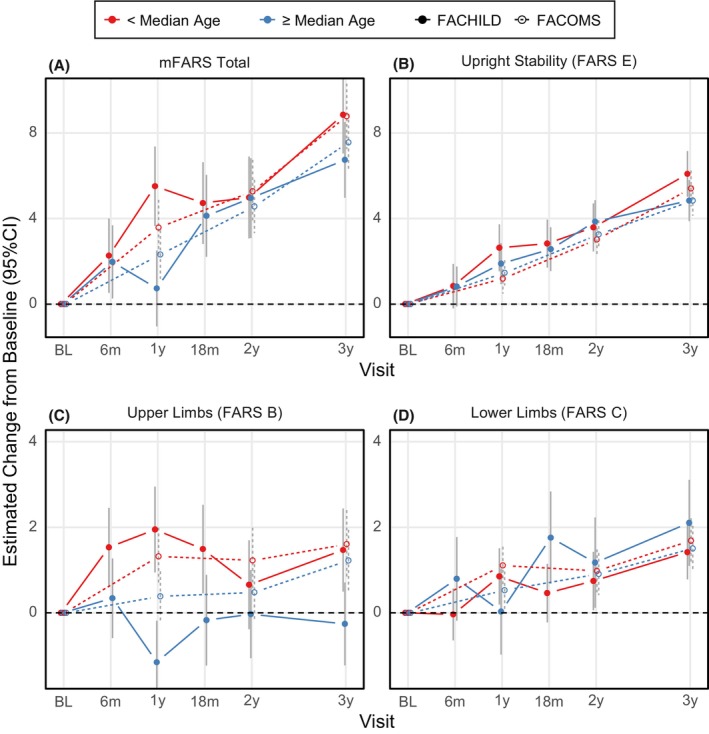
mFARS changes from baseline by study and age‐based subgroups: Below (red) and above or equal (blue) median age (13.1 years). Results from FACHILD (solid dots and lines) and FACOMS (circles, dotted lines).

**Figure 5:**
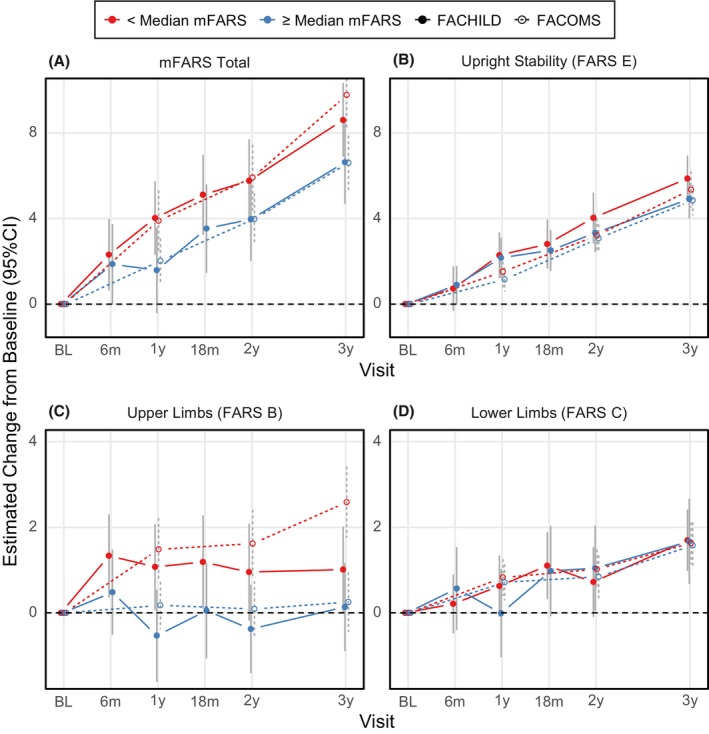
mFARS changes from baseline by study and mFARS‐based subgroups: Below (red) and above or equal (blue) Median mFARS Score (37). Results from FACHILD (solid dots and lines) and FACOMS (circles, dotted lines).

**Figure 6:**
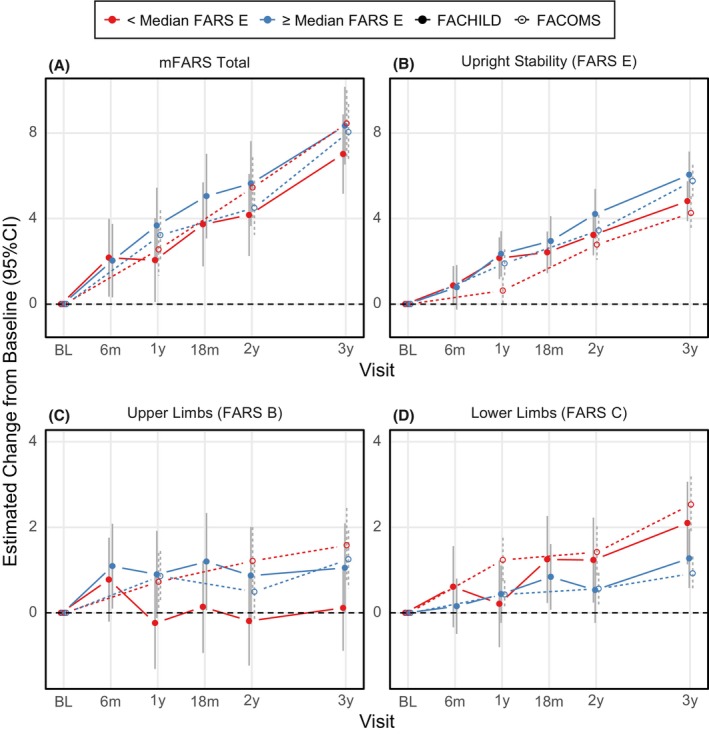
mFARS changes from baseline by study and FARS E‐based subgroups: FARS E Results below (red) and above or equal (blue) Median FARS E Score (22.3).

Next, the cohorts were analyzed within relevant subgroups (by median age, baseline mFARS and FARS E, respectively). Results from these groups indicated FARS E scores as remarkably consistent (Figs. 4, 5, 6). Differences between subgroups arose for mFARS and were typically driven by differences in FARS B, and to a smaller extent by FARS C.

As an example, younger patients in FACHILD (red group, Fig. 4C) declined by 1.9 points in FARS B within the first year vs. the older group improving by −1.2 points. At later timepoints, changes stabilized with high variability. When split by median mFARS scores, the less severe group (blue group, Fig. 5C) showed little FARS B decline overall (3‐year result +0.1 points in both studies), while the more severe group declined by 1.0 points in FACHILD and 2.5 points in FACOMS. This was the main cause for mFARS scores to also differ between the less and more severely affected groups (below and above median mFARS). The group with lower mFARS values at baseline declined notably faster (8.6 points, 95% CI 6.9 to 10.3 vs 6.6 points, 95% CI 4.7 to 8.6) at 3 years. In this case, results were confirmed by FACOMS data.

When assessing consistency of decline over the 3 years study period, FARS B was clearly the least consistent subscore, apparent from all three subgroup splits. FARS C subscore results were more regular over the subgroups than of FARS B, but also with more variability than FARS E. In general, from visual assessment, subgroups by median FARS E (Fig. 6) showed the most consistent behavior, indicating that FARS E might be the overall best predictor of disease progression. FACOMS results (294 individuals vs 89 in FACHILD) showed less variability, and the subgroups behaved more similarly.

### Impact of baseline covariates

To explore the impact of potential baseline covariates (baseline score, baseline age, age at onset, and GAA1 repeat length), individual models were fitted for mFARS and FARS E, independently for FACHILD and FACOMS. For FACHILD (*n* = 89), only the baseline score showed a statistically significant impact on change from baseline, and only for the mFARS model. With the larger sample size of the FACOMS group (*n* = 294), GAA1 also became statistically significant for FARS E (not for mFARS). Overall, this shows a minor influence of genetic and onset parameters on these models. A summary of model results was provided in Table S3.

## Discussion

The FACHILD natural history study was designed to expand the knowledge about the pediatric patient population, and to assess change over time more frequently, similar to randomized controlled trials (RCTs). Individuals from all ambulatory stages and diverse genetic and clinical severities were recruited. This report focused on the effectiveness of the mFARS and its subscores in ambulatory children. In adults, the scale is a sensitive and relevant tool to assess neurological disease progression.^8^, ^9^

Baseline mFARS scores correlated weakly with age, indicating that all disease stages exist at nearly all ages in a pediatric FRDA population. This further illustrates the challenges in these very fast progressing patients, diagnosed at early ages. It also underlines the requirement to demonstrate that the tools used for assessing these patients function consistently over the target population.

The overall cohort changes in mFARS score confirmed that the scale can function in children; these results were consistent with complete FACOMS data^4^, and clinical trials enrolling children.^10^, ^11^ There was also a notable consistency between patients participating in FACHILD and the comparative FACOMS cohort, taken into account that the latter cohort was enrolled over the last 2 decades. This was most evident in the FARS E (Upright Stability) subscore, the main driver of progression in ratings scales^4^ and, with regard to consistency and variability, also for FARS C (lower limb coordination). On the other hand, and not limited to specific subgroups, results in upper limb scores (FARS B) showed higher variability.

Over all 3 years, FARS E progressed slightly faster in FACHILD, while FARS B progressed slower in this group; This was counterintuitive, considering the slightly higher genetic severity in FACHILD (e.g., median age of onset 7 vs 8).

Subgroup analyses allowed deeper insight into the patterns of progression. These analyses are designed to assess potential heterogeneities and to indicate confounders of progression. Subgroups by median baseline age behaved very consistently in FARS E, but not in FARS B. This situation was very similar in subgroups split by median baseline mFARS and median baseline FARS E. Importantly, FACHILD vs FACOMS results were closer together than the respective subgroups by age, and by baseline values. Overall, instability in FARS B drove inconsistent mFARS results.

Besides the sample size, the main difference between both studies was the 6‐month interval visits in FACHILD. While designed to resemble trials more closely, this change might have, unintentionally, revealed issues with FARS B that could be of high relevance for future trials.

Evident from the age‐based subgroup results, younger participants in both FACHILD and FACOMS progressed faster than older ones and this effect was much more dramatic in the smaller FACHILD cohort. After 2 years, the group results coalesce again, with a pattern potentially indicating convolution of potential learning effects with faster progression in younger, genetically more affected individuals. A learning effect compensating for disease progression can also be found from subgroups based on median mFARS. Especially in FARS B (Fig. 5C), lower baseline scores progress faster, but in FACHILD these scores stay constant at 1 year and thereafter, while FACOMS patients continue to decline.

Taken together, these results reveal problematic properties of FARS B that might be exacerbated when repeated visits occur even more frequently (as in clinical trials for higher statistical power). Potential training effects not only might have increased variability but also decreased the extent of decline. In addition, placebo effects occur in these appendicular items,^8^ likely adding to variability. On the other hand, while FARS B has only a minor contribution to overall decline in NH studies in the discussed population, it can be affected by interventions: It was the only mFARS subscore that in itself showed a statistically significant difference^12^ between treatment groups in the MOXIE study and it contributed substantially to the overall treatment effect providing pivotal evidence to the approval of omaveloxolone for the treatment of adults with FRDA. Such findings warrant thorough investigation in the future and they will not be limited to mFARS, but also other ataxia rating scales relying on similar functional tests.

All these considerations indicate that FARS E may be the best descriptor of disease progression in children. It is the main driver of decline in ambulant patients in NH studies^4^, ^13^, behaves most consistent over time, and its progression shows less variability vs FARS B when comparing relevant subgroups. Also, the items in FARS E all have direct clinical meaning as they generally replicate functions from daily life. The individual balance items tested in FARS E are lost sequentially, describing the nature of function loss in FRDA, and as such are predictive for the time to loss of ambulation, a highly relevant and meaningful milestone.^14^ The direct clinical significance in upper limb scores is less obvious, and reproducibility issues of ataxias scale are frequently attributed to upper extremity items.^15^

It is less clear, however, how the benefits of FARS E over total mFARS would impact sample sizes, particularly because treatment effects on upper limbs, although of less clear relevance, cannot be detected and placebo effects are hard to predict. In our study, the simplistic approach of standard response means indicated that the effects found for FARS B, induced potentially by more frequent visits in FACHILD, render FARS E the clearly favorable outcome measure.

Other factors add complexity to trials in FRDA, like the potential impact of demographic and genetic factors such as the GAA1 repeat length, age of onset, and age at baseline. These are all interconnected and impact the rates of change over time. This impact, however, will not be detected in studies with typical RCT sizes (such as FACHILD).

Another factor is the presence of individuals compound heterozygous for the *FXN* mutations, which are often excluded from participation in RCTs. Our results suggest that FARS E could cover a more inclusive population with regard to the relatively frequent G130V mutation, which causes a unique phenotype sparing arms and bulbar function. Such rare patients might only lead to overall increases in variability in the data in FARS B.

Several studies have discussed the problems arising from the application of rating scales in young children,^4^, ^15^ and we confirm a danger of reduced sensitivity with mFARS as a primary outcome in this population. There are also signs that FARS B can impact the results beyond additional variability. Conversely, FARS E alone may provide a suitable measure across all pediatric ages, as well as early and late ambulatory disease stages. On the other hand, the total score should function well beyond ages when maturation of motor coordination may have been completed. Notably, a different relationship between FARS B and E over various age groups suggests that the typical inclusion criterion for clinical studies (mFARS <20) should be revisited.

One main limitation of this study was the impact of the COVID‐19 pandemic, although the intermittent data loss of mFARS data seemed manageable. The acquired data were very complete, and FACOMS results were helpful to contextualize the results. In general, the retention of individuals for 6‐month intervals between visits was successfully accomplished, although it proved challenging for a NH study. The use of virtual visits was beneficial in the present study, but of limited value for measures such as the mFARS. Their use may be warranted in specific situations in future studies, including clinical trials.

Clinical trials in FA are challenging, more so in children, as genetic disease severity leads to early occurrence of symptoms simultaneously with early individual development and puberty, resulting in complex effects on neurological progression. Progression is further complicated by features of scoliosis and occasionally cardiac disease in children, requiring assessment of such comorbidities in study interpretation. The present study provides a well‐defined cohort for continued follow‐up to define the evolution of disease in children with attention to such comorbidities. Results from additional outcome measures, such as ADL scores, timed walks (25 feet and 1 minute), the Berg Balance Scale, and frataxin levels will be reported in due course.

## Author Contributions

CR conceived and designed the work, analyzed and interpreted the data, and drafted the article; SP, revised the article critically and collected the data; SHS, revised the article critically and collected the data; JF, conceived the study and revised the article critically; DRL conceived the study, designed the work, analyzed and interpreted the data, drafted the article, and collected the data.

## Funding Information

This work was funded by the Friedreich's Ataxia Research Alliance (FARA) and *an FDA R01 Grant*.

## Conflict of Interest

CR reports consultancy fees from FARA, The National Ataxia Foundation, Reata, PTC, Biohaven, Takeda, Santhera, and Solid Biosciences; DRL receives (outside the present study) grants from the NIH, FDA, FARA, MDA, Design, Retrotope, PTC, Reata, Stealth, Voyager, and Novartis. The remaining authors report no competing interests.

## Supporting information


Appendix S1.

## Data Availability

Data will be made part of the Friedreich's Ataxia Integrated Clinical Database (FA‐ICD), which is available on appropriate request via the Rare Diseases Cures Accelerator Data and Analytic platform (RDCA‐DAP) at the Data Collaboration Centre (DCC) of the Critical Path Institute (C‐Path).
