# Supplementary material for: Evaluating mFARS in pediatric Friedreich's ataxia: Insights from the FACHILD study
Source: Ann Clin Transl Neurol. 2024 Mar 31;11(5):1290–300. doi: 10.1002/acn3.52057 (PMC11093230; doi:10.1002/acn3.52057)
Supplement: Supplementary file 1 — Appendix S1. [file ACN3-11-1290-s001.docx]

# Supplementary Material

Supplementary Figure 1: Impact of the COVID Pandemic: Overview of Follow-up Visits, including virtual visits. Visits are colored by Site. The dashed line marks last-patient first visit (LPFV), just before March 11^st^ 2020, the official start of the COVID pandemic.


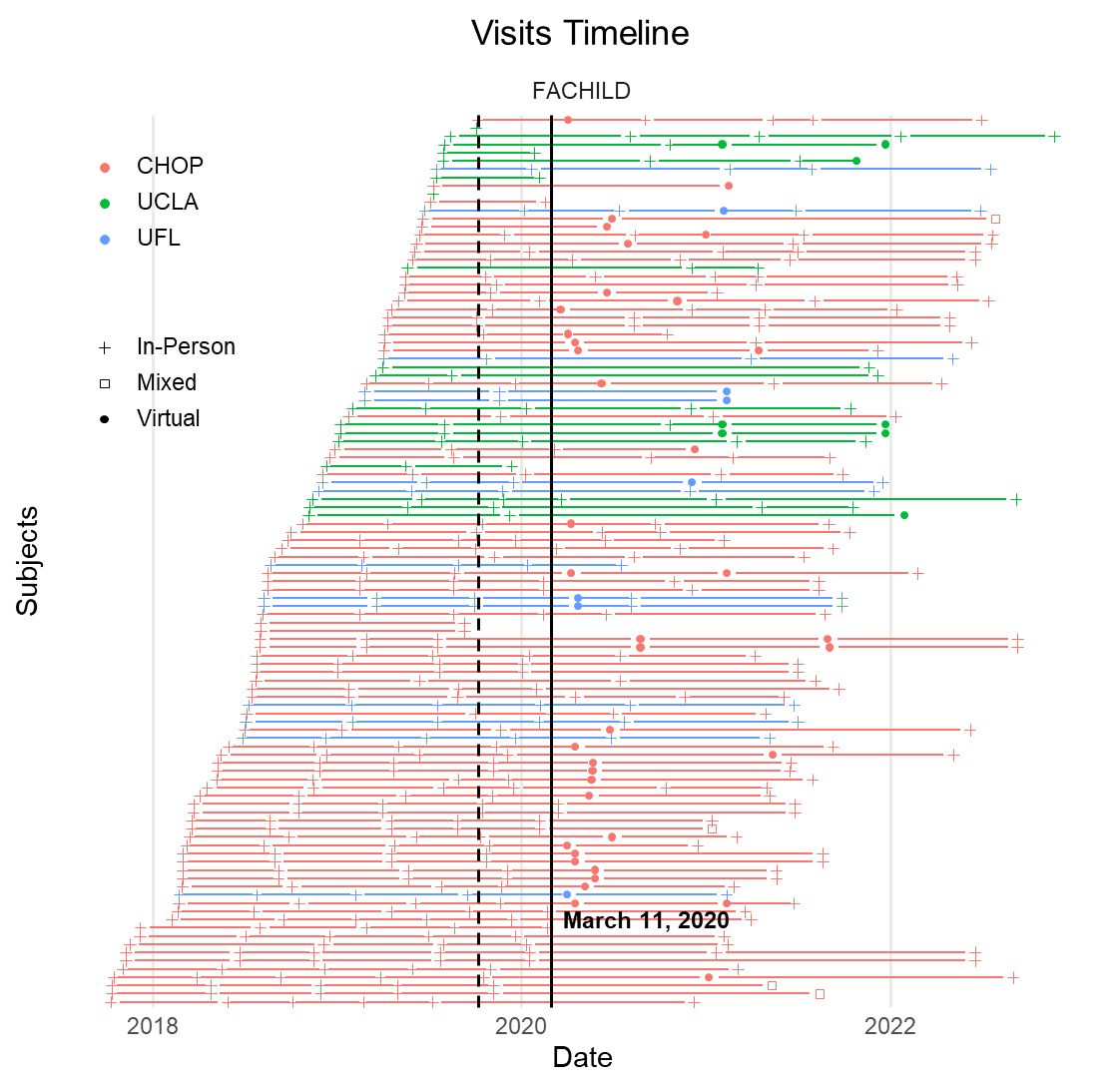


Supplementary Table 1: Demographic Summary of the all enrolled population: Others includes missing mFARS data early disease stage (Baseline mFARS < 17).

|  | Analysis Population | Other  (ambulatory) | Non-ambulatory |
| --- | --- | --- | --- |
| n | 89 | 6 | 13 |
| Sex (male) | 41 (46.1) | 4 (66.7) | 5 (38.5) |
| Age | 13.3 (2.8) | 13.6 (3.0) | 16.0 (1.7) |
| Age of Onset | 7.0  [5.0, 10.0] | 8.0  [6.2, 12.0] | 6.0  [5.0, 7.0] |
| GAA1 | 766.0  [699.0, 893.8] | 805.0  [605.0, 1072.5] | 775.0  [637.5, 866.0] |
| Compound Heterozygotes | 8 (9.0) | 1 (16.7) | 1 (7.7) |
| mFARS Total | 37.8 (11.7) | 21.0 (20.2) | 63.4 (7.5) |
| Upright Stability (E) | 22.3 (5.3) | 11.9 (10.5) | 34.2 (0.9) |
| Upper Limbs (B) | 9.2 (5.3) | 5.4 (6.5) | 14.9 (4.1) |
| Lower Limbs (C) | 5.9 (2.4) | 3.2 (2.8) | 13.5 (3.0) |
| Bulbar Function (A) | 0.3 (0.5) | 0.5 (0.7) | 0.9 (0.5) |
| Data are n (%), median [IQR] or mean (SD) | | | |

Supplementary Table 2: Numerical Results for Total mFARS, FARS E and B Sub Scores in the overall FACHILD and FACOMS cohorts.

| Study | Scale | Visit | Estimate (Change from Baseline) | 95%CI | p-value | Sign. | SRM |
| --- | --- | --- | --- | --- | --- | --- | --- |
| FACHILD | mFARS | 1y | 2.9 | (1.6, 4.2) | <0.0001 | *** | 0.60 |
| FACHILD | mFARS | 2y | 4.9 | (3.6, 6.3) | <0.0001 | *** | 0.91 |
| FACHILD | mFARS | 3y | 7.7 | (6.4, 9.0) | <0.0001 | *** | 1.15 |
| FACHILD | FARS.E | 1y | 2.2 | (1.5, 2.9) | <0.0001 | *** | 0.82 |
| FACHILD | FARS.E | 2y | 3.7 | (3.0, 4.5) | <0.0001 | *** | 1.16 |
| FACHILD | FARS.E | 3y | 5.5 | (4.7, 6.2) | <0.0001 | *** | 1.58 |
| FACHILD | FARS.B | 1y | 0.3 | (-0.4, 1.1) | 0.3726 |  | 0.13 |
| FACHILD | FARS.B | 2y | 0.3 | (-0.4, 1.1) | 0.3959 |  | -0.02 |
| FACHILD | FARS.B | 3y | 0.6 | (-0.1, 1.3) | 0.1052 |  | 0.14 |
| FACOMS | mFARS | 1y | 2.9 | (2.0, 3.7) | <0.0001 | *** | 0.42 |
| FACOMS | mFARS | 2y | 4.9 | (3.9, 5.8) | <0.0001 | *** | 0.72 |
| FACOMS | mFARS | 3y | 8.1 | (7.1, 9.1) | <0.0001 | *** | 1.04 |
| FACOMS | FARS.E | 1y | 1.3 | (0.9, 1.8) | <0.0001 | *** | 0.34 |
| FACOMS | FARS.E | 2y | 3.1 | (2.6, 3.7) | <0.0001 | *** | 0.85 |
| FACOMS | FARS.E | 3y | 5.1 | (4.6, 5.6) | <0.0001 | *** | 1.22 |
| FACOMS | FARS.B | 1y | 0.8 | (0.4, 1.3) | 0.0004 | *** | 0.19 |
| FACOMS | FARS.B | 2y | 0.8 | (0.3, 1.3) | 0.0018 | ** | 0.27 |
| FACOMS | FARS.B | 3y | 1.4 | (0.8, 1.9) | <0.0001 | *** | 0.37 |

Supplementary Table 3: Impact of Baseline Covariates

| Study | Parameter | effect | term | estimate | std.error | statistic | df | p.value | conf.low | conf.high |
| --- | --- | --- | --- | --- | --- | --- | --- | --- | --- | --- |
| FACHILD | FARS.E | fixed | Baseline Score | -0.097 | 0.061 | -1.598 | 80.004 | 0.114 | -0.217 | 0.024 |
| FACHILD | FARS.E | fixed | Age at Baseline | -0.001 | 0.134 | -0.004 | 82.832 | 0.996 | -0.267 | 0.266 |
| FACHILD | FARS.E | fixed | Age of Onset | -0.109 | 0.112 | -0.974 | 81.609 | 0.333 | -0.332 | 0.114 |
| FACHILD | FARS.E | fixed | GAA1 | 0.219 | 0.199 | 1.099 | 77.076 | 0.275 | -0.178 | 0.615 |
| FACHILD | mFARS | fixed | Baseline Score | -0.109 | 0.047 | -2.329 | 81.374 | **0.022** | -0.202 | -0.016 |
| FACHILD | mFARS | fixed | Age at Baseline | -0.183 | 0.220 | -0.832 | 81.722 | 0.408 | -0.619 | 0.254 |
| FACHILD | mFARS | fixed | Age of Onset | -0.287 | 0.187 | -1.536 | 79.988 | 0.128 | -0.658 | 0.085 |
| FACHILD | mFARS | fixed | GAA1 | 0.226 | 0.327 | 0.691 | 75.094 | 0.492 | -0.425 | 0.877 |
| FACOMS | FARS.E | fixed | Baseline Score | -0.172 | 0.042 | -4.113 | 276.193 | **0.000** | -0.255 | -0.090 |
| FACOMS | FARS.E | fixed | Age at Baseline | 0.125 | 0.097 | 1.293 | 268.972 | 0.197 | -0.065 | 0.316 |
| FACOMS | FARS.E | fixed | Age of Onset | -0.171 | 0.097 | -1.771 | 272.102 | 0.078 | -0.361 | 0.019 |
| FACOMS | FARS.E | fixed | GAA1 | 0.429 | 0.134 | 3.193 | 281.066 | **0.002** | 0.164 | 0.693 |
| FACOMS | mFARS | fixed | Baseline Score | -0.182 | 0.042 | -4.355 | 280.227 | **0.000** | -0.264 | -0.100 |
| FACOMS | mFARS | fixed | Age at Baseline | 0.010 | 0.183 | 0.055 | 271.784 | 0.956 | -0.351 | 0.371 |
| FACOMS | mFARS | fixed | Age of Onset | -0.368 | 0.188 | -1.955 | 272.009 | 0.052 | -0.738 | 0.003 |
| FACOMS | mFARS | fixed | GAA1 | 0.468 | 0.256 | 1.828 | 281.268 | 0.069 | -0.036 | 0.971 |
